# Mild magnetic nanoparticle hyperthermia enhances the susceptibility of *Staphylococcus aureus* biofilm to antibiotics

**DOI:** 10.1080/02656736.2019.1707886

**Published:** 2020

**Authors:** Layla Alumutairi, Bing Yu, Mitchell Filka, Joseph Nayfach, Min-Ho Kim

**Affiliations:** a School of Biomedical Sciences, Kent State University, Kent, OH, USA; b Department of Biological Sciences, Kent State University, Kent, OH, USA; c Qteris, Inc, San Rafael, CA, USA; d Department of Biology, Princess Nourah bint Abdulrahman University, Riyadh, Saudi Arabia

**Keywords:** Magnetic nanoparticles, alternating magnetic field, hyperthermia, antibiotics, *S. aureus* biofilm

## Abstract

**Objective:**

A critical challenge in the treatment of biofilm infection is the capacity of biofilm-grown bacteria to develop resistance to traditional antimicrobial therapies. The objective of this study was to validate the therapeutic potential of magnetic nanoparticle/alternating magnetic field (MNP/AMF) hyperthermia in combination with conventional antibiotics against biofilm infection.

**Materials and methods:**

The impact of MNP/AMF hyperthermia on the viability of S. aureus biofilm in the absence and presence of antibiotics as well as on the bactericidal activity of macrophages were evaluated at varying conditions of MNPs concentration and AMF intensity using in vitro cell culture models.

**Results:**

The application of MNP/AMF alone at a CEM43 thermal dose below the threshold for skin tissue exhibited a modest efficacy in the eradication of Staphylococcus aureus (S. aureus) biofilm (<1-log reduction). The treatment of antibiotics (ciprofloxacin, vancomycin) alone at a bactericidal concentration for planktonic S. aureus had no significant effect on the eradication of biofilm phase of S. aureus. However, when the biofilm was pre-exposed to mild MNP/AMF hyperthermia, the treatment of antibiotics could exhibit bactericidal effects against S. aureus biofilm, which was associated with increased uptake of antibiotics to the bacterial cells. Importantly, the application of MNP/AMF could promote the bactericidal activity of macrophages against intracellular bacteria via MNP-dependent generation of reactive oxygen species (ROS).

**Conclusion:**

Our results validate that the application of mild MNP/AMF hyperthermia within a safe thermal dose threshold is synergistic with conventional antibiotics as well as aids host innate immune response of macrophages for the clearance of intracellular bacteria.

## Introduction

A growing body of evidence suggests that the formation of bacterial biofilm plays a significant role in the pathogenesis of wound chronicity [1–5]. A critical challenge in the treatment of biofilm infection is the capacity of biofilm bacteria to develop resistance to traditional antibiotics therapies. The molecular mechanisms of biofilm resistance have been associated with slow or incomplete penetration of antimicrobials to bacterial cells due to the presence of biofilm matrix [6], decreased metabolism of bacteria in the biofilm phase, and the induction of a biofilm-specific bacterial phenotype that results in the expression of genes to interfere with the actions of antimicrobial agents [7,8]. Despite the development of novel antimicrobial agents, the cost and complexity of treating chronic wounds associated with biofilm infections remain a serious challenge.

We previously developed a magnetic nanoparticle (MNP) hyperthermia platform and validated its capacity to target and substantially reduce the viability of bacterial pathogens using an *in vivo* mouse model of *Staphylococcus aureus* (*S. aureus*) wound infection [9]. The principle of this method is to induce a localized increase in temperature in bacterial cells by targeted activation of MNPs with an externally applied energy source of high frequency alternating magnetic field (AMF) [10–12]. However, the use of MNP/AMF hyperthermia alone is still limited due to potential thermal damage to the host tissue, since it typically requires the application of MNP/AMF at higher thermal doses for complete eradication of bacterial pathogens.

It has been shown that the application of heat shock to the biofilm phase of bacteria by means of uniformly increasing temperature in the culture media could significantly enhance the susceptibility of *Staphylococcal* as well as *Pseudomonas aeruginosa* (*P. aeruginosa*) biofilm to conventional antibiotics [13,14]. Furthermore, the application of AMF on *P. aeruginosa* biofilm formed on metallic implants rendered the bacteria more susceptible to antibiotics *via* heat-mediated removal of the biofilm extracellular polymeric substances (EPS) [15]. Although these studies were associated with the application of heat shock at high thermal doses, they validate the proof-of-principle for hyperthermia therapy in rendering biofilm bacteria more sensitive to conventional antibiotics. Given the promise of MNP/AMF hyperthermia to non-invasively generate heat shock within the bacterial cells, we have reasoned that a combined treatment with conventional antibiotics would overcome the current limitation of MNP/AMF hyperthermia for clinical translation.

The objective of this study was to validate the therapeutic potential of MNP/AMF hyperthermia in combination with conventional antibiotics against biofilm infections. To address this, we have evaluated the impact of MNP/AMF hyperthermia on the elimination of biofilm bacteria in the absence and presence of antibiotics by using an *in vitro* model of *S. aureus* biofilm, a major human pathogen implicated in chronic wound infections [16]. Given the physiological importance of host innate immune response to bacterial infections, we have also examined the effect of MNP/AMF hyperthermia on the bactericidal activity of macrophages. Our findings demonstrated that the application of MNP/AMF hyperthermia at a safe thermal dose could be successfully combined with conventional antibiotics against biofilm infection.

## Materials and methods

### Preparations of the planktonic and biofilm culture of S. aureus

*S. aureus* (ATCC 6538 strain) was streaked onto the tryptic soy agar (TSA) plate (Difco™, BD). A single colony of *S. aureus* was inoculated to the 5 ml of tryptic soy broth (TSB) (BD Bacto) and incubated overnight under aerobic conditions at 37 °C in a shaker at 180 rpm. For the preparation of planktonic *S. aureus*, the cells were pelleted by centrifugation at 3,500 g and 4 °C for 7 min and diluted with TSB to obtain the desired concentrations of bacteria (1 × 10^6^–2 × 10^8^ CFU/mL). For the culture of *S. aureus* biofilm, 1 × 10^6^ CFU/mL of stationary growth phase *S. aureus* was added to the wells of a sterile polystyrene 8-well chamber slide or a 48-well polystyrene plate with TSB with 1% glucose (TSBG), and incubated for 48 h at 37 °C under static conditions. This resulted in the proliferation of bacteria from 1 × 10^6^ to 2 × 10^8^ CFU/mL.

### Flow cytometry

Varying concentrations of MNPs (0.1–1 mg/mL, 100 nm in size, nanomag-D-SPIO, Micromod GmBH) were added to the wells of *S. aureus* biofilm and incubated for 2 h. The biofilm was then disrupted by placing the wells in a sonication bath (Bransonic CPX1800H) for 10 min. The cells were pelleted by centrifugation, suspended with phosphate-buffered saline (PBS), and analyzed by flow cytometry (Accuri C6, BD Biosciences). The change in side-scattering patterns of the bacterial cells was detected.

### Atomic absorption spectroscopy (AAS) measurement for the quantification of iron levels in bacterial cells

The internalization of MNPs by *S. aureus* was assessed by quantifying iron levels in the bacteria using an AAS. Briefly, *S. aureus* (1 × 10^8^ CFU/mL) were incubated with MNPs (1 mg/mL) for 2 h and then pelleted by centrifugation. The pellet was washed twice with PBS and digested with 75% HNO_3_ to destroy the organics, and the metal ions in the cells were then converted to oxides by calcination of the sample. The metal oxides obtained were dissolved in aqua regia and the concentration of iron in the solution was analyzed using an AAS.

### Determination of the susceptibility of planktonic and biofilm phase of S. aureus to antibiotics

The susceptibility of planktonic and biofilm phase of *S. aureus* to antibiotics were determined by using the macrodilution broth method [17]. To determine the efficacy of antibiotics against planktonic *S. aureus*, either ciprofloxacin (CIP, Sigma-Aldrich) or vancomycin (VAN, Sigma-Aldrich) prepared at concentrations ranging from 0.1 µg/mL to 64 µg/mL was treated to the planktonic *S. aureus* (2 × 10^8^ CFU/mL) for 18 h at 37 °C. The cells were then plated on TSA for bacterial CFU counting. The susceptibility of *S. aureus* biofilm to the antibiotics was determined by treating varying concentrations of CIP or VAN (ranging from 0.1 µg/mL to 1,024 µg/mL) to the wells of established *S. aureus* biofilm for 18 h at 37 °C. The cells were then plated on TSA for bacterial CFU counting after disrupting biofilm matrix by placing the wells in a sonication bath for 10 min.

### Measurements of temperature increase (ΔT) during the exposure of MNP/AMF hyperthermia

The procedure for the application of MNP/AMF hyperthermia to the biofilm was performed as described in our previous study [9]. In brief, varying concentrations of MNPs ranging from 1 to 3 mg/mL were added to the wells of *S. aureus* biofilm pre-formed on a polystyrene 8-well chamber slide and incubated for 2 h. For the application of AMF, the chamber slide was inserted in a water-cooled magnetic coil and an AMF was applied for 6 min at varying field strengths of 18, 24, and 30 kA/m at a frequency of 2.1 MHz. The real-time monitoring of temperature in the culture solution during the AMF exposure was performed using a fiber optic temperature probe (Neoptix) placed in the solution.

### Determination of cumulative equivalent minutes at 43 °C (CEM_43_)

The CEM_43_ was calculated from equation as described [18], CEM_43_ = ∑ t_*i*_ ×R^(43−T)^, where T is the average temperature during *i*-th time interval t_*i*_ (min), and R is a constant equal to 0.25 for *T* < 43 °C and 0.5 for *T* > 43 °C. The average temperature T was determined by a plot of ΔT vs time measured during the application of MNP/AMF hyperthermia.

### S. aureus biofilm viability assay using SYTO 9-propidium iodide (PI) staining

A SYTO9-PI biofilm viability kit (FilmTracer™ LIVE/DEAD, Thermo Fisher Sientific) was used to assess the effect of MNP/AMF hyperthermia on the viability of *S. aureus* biofilm. In brief, 200 µL of fluorescent staining solution made of SYTO9 and PI mixture was added to the MNP/AMF-treated *S. aureus* biofilm on an 8-well chamber slide. The biofilms were incubated for 20 min in the dark at room temperature, fixed with 200 µL of neutral buffered formalin after washing out the staining solution, and then imaged under an Olympus IX81 fluorescence microscope. The live bacteria (SYTO9) were seen with a 490 nm excitation and 535 nm emission filter and dead bacteria (PI) were seen with a 490 nm excitation, 635 nm emission filter.

### Antibiotic susceptibility of S. aureus biofilm in the presence of MNP/AMF hyperthermia

The *S. aureus* biofilm pre-formed on an 8 well-chamber slide was incubated with MNPs (1–3 mg/mL) for 2 h at 37 °C and then applied with an AMF for 6 min at varying field strengths (18, 24, and 30 kA/m). Following the application of MNP/AMF, the biofilms were treated with CIP or VAN at a concentration of 16 µg/mL for 18 h at 37 °C and then bacterial CFUs were counted as described above.

### Penetration of antibiotics through biofilms

The penetration of antibiotics through the biofilms was determined according to the method described previously [19]. Briefly, *S. aureus* biofilm were grown on 6 mm polycarbonate membrane overnight and a 6 mm nitrocellulose membrane (pore size of 0.4 µm) was placed on the surface of biofilm. In a separate group of biofilms, the polycarbonate membrane with *S. aureus* biofilm was applied with MNP/AMF at 2 mg/mL of MNPs and 30 kA/m of AMF for 6 min and then nitrocellulose membrane was placed on the surface of biofilm. Then, an antibiotic disk with CIP at concentrations of 16, 32, 64, or 128 µg/mL was placed on the top of the membrane-bound biofilm with or without MNP/AMF treatment. The biofilms were transferred to the TSA plate inoculated with planktonic phase of *S. aureus*. The TSA plate was incubated overnight at 37 °C and the diameter of the zone of growth inhibition were measured.

### Quantification of antibiotic uptake by planktonic and biofilm S. aureus

The uptake of antibiotics by bacterial cells was evaluated using Bodipy-FL-vancomycin (FL-VAN, Invitrogen), fluorophore conjugated vancomycin. For this, either planktonic or biofilm phase *S. aureus* was treated with FL-VAN at a sub-lethal dose of 0.1 µg/mL for a given duration, at which the viability of *S. aureus* was not altered. The antibiotic uptake was quantified by detecting fluorescence emitted from FL-VAN at 510 nm using a spectrophotometer (SpectraMax® M4 Multi-Mode Microplate Reader, Molecular Devices). To assess the effect of MNP/AMF hyperthermia on the uptake of antibiotics by biofilm bacteria, *S. aureus* biofilm was pretreated with MNPs (1 and 2 mg/mL) for 2 h and then applied with an AMF for 6 min at 30 kA/m. Cells were then incubated with 0.1 µg/mL FL-VAN for between 1 and 24 h and the fluorescence from FL-VAN was measured. The extent of antibiotic uptake was expressed after normalizing the fluorescence intensity with respect to the number of viable bacteria in the well.

### Cytotoxicity of MNP/AMF on RAW 264.7 macrophages

RAW 264.7 cells (ATCC, Manassas, VA) were cultured in complete DMEM, and supplemented with glutamine (2 mM), penicillin (100 U/ml), streptomycin sulfate (100 mg/ml), and 10% fetal bovine serum (FBS). Cells (40,000 cells per well) were then seeded into each well of a 96-well plate for 24 h. To determine the cytotoxicity of MNP/AMF hyperthermia on RAW 264.7 cells, varying concentrations (0–3 mg/mL) of MNPs were treated to the wells with RAW 264.7 cells for 2 h. Then, the cells were applied with an AMF for 6 min at a field strength of 30 kA/m. To determine cytotoxicity, 10 µL of MTT solution (Trevigen) was added to each well of the microtiter plate for an additional 2 h and absorbance values were measured at 570 nm using a spectrophotometer.

### Antibiotic protection assay for assessing the bactericidal activity of macrophages

To assess the effect of MNP/AMF on the bactericidal activity of macrophages against intracellular bacteria, RAW 264.7 cells were seeded in a 96-well plate at a density of 70,000 cells per well for 24 h. Then, cells were pretreated with MNPs (1 and 2 mg/mL) for 2 h, and applied with an AMF for 6 min at a field strength of 30 kA/m. The cells were then incubated with planktonic *S. aureus* at a 1:10 multiplicity of infection at 37 °C for 1 h and washed with PBS to remove non-adherent bacteria. The cells were treated with a serum-free Dulbecco’s Modified Eagle’s Medium (DMEM) with 25 µg/mL gentamicin (Sigma-Aldrich) for 18 h in order to kill and remove the extracellular bacteria and then lysed with PBS containing 0.1% Triton X-100 at room temperature for 10 min. The cell lysates were serially diluted with sterile PBS and 50 µL of each dilution was plated on triplicate tryptic soy agar plates. The plates were incubated overnight at 37 °C. The number of surviving bacteria within RAW 264.7 cells was determined by CFU counting.

### Measurement of the levels of intracellular reactive oxygen species (ROS) in RAW 264.7 macrophages

The level of intracellular ROS was quantified using 5-(and-6)-carboxy-2′,7′-dichlorodihydrofluorescein diacetate (carboxy-H2DCFDA, Thermo Fisher Scientific). To assess the effect of MNP/AMF hyperthermia on reactive oxygen species (ROS) generation in macrophages, RAW 264.7 cells were seeded at a density of 40,000 cells per well in a 96-well plate and incubated with MNPs (0–2 mg/mL) for 2 h, followed by treatment with an AMF at 30 kA/m for 6 min. The cells were incubated with Dulbecco’s PBS (DPBS) containing 25 µM carboxy-H2DCFDA for 30 min at 37 °C for the detection of ROS. Following the incubation, RAW 264.7 cells were washed twice to remove excessive fluorescent dye, and the fluorescence intensity was quantified using a spectrophotometer.

### Statistical analysis

Data analysis was performed using an Origin 2015 software (Origin Lab). A two-tailed unpaired t-test was used to determine statistical significance between two groups. Statistical tests among multiple groups were analyzed using one-way ANOVA followed by Turkey’s posttest for secondary analysis for comparison. For all analyses, *p*-values of less than .05 was considered to be statistically significant. Data were presented as mean ± standard error (SE).

## Results

### The MNP/AMF hyperthermia-induced increase of CEM_43_ was dependent on MNPs concentration and AMF intensity

**Figure 1.**
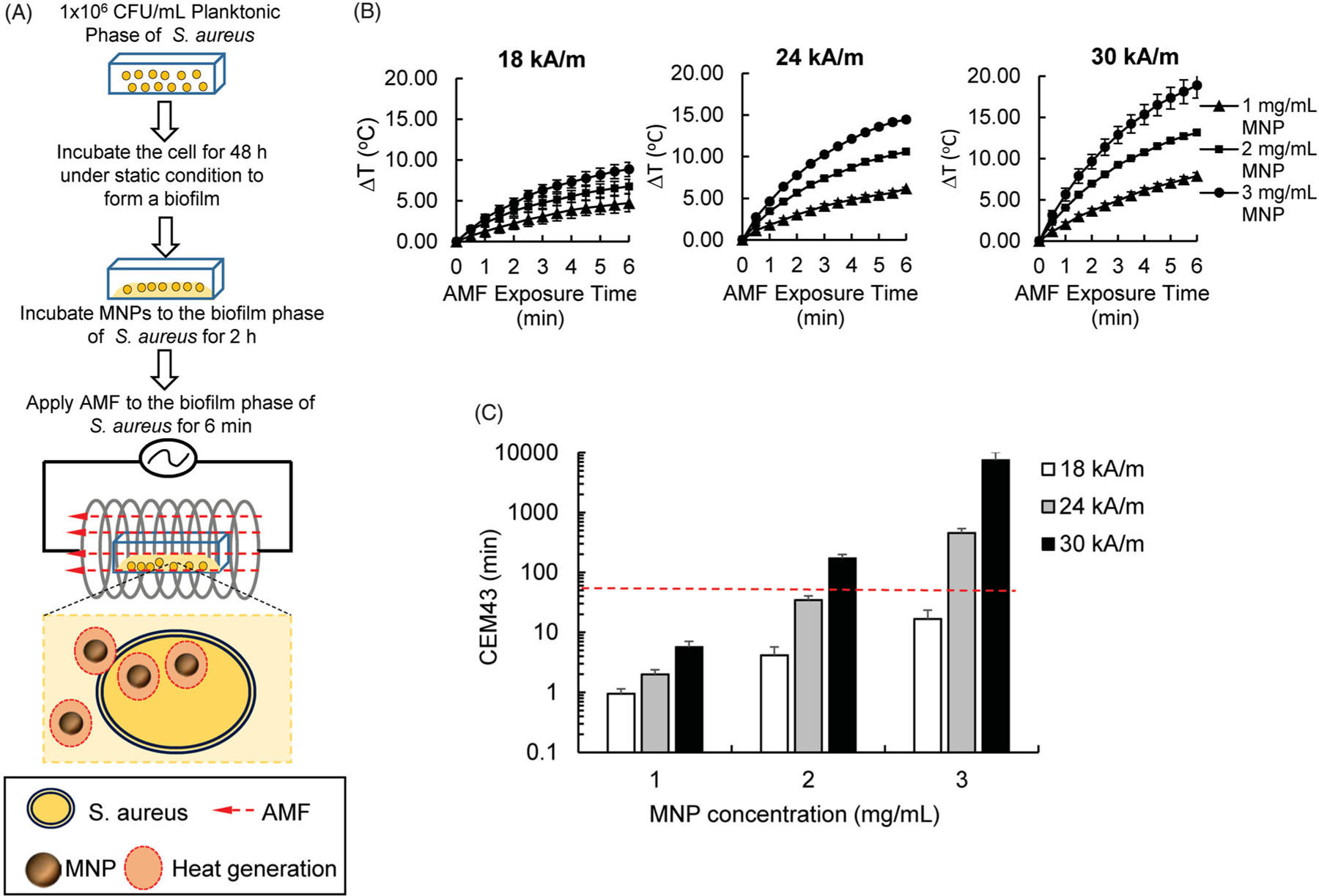
Effects of MNP/AMF hyperthermia on the CEM_43_ thermal dose for varying conditions of MNPs concentration and AMF intensity. (A) A schematic diagram on the experimental procedure for the application of MNP/AMF hyperthermia to the biofilm bacteria *in vitro*. (B) Increase in temperature (ΔT) during the application of MNP/AMF as a function of MNP concentration (1–3 mg/mL) and AMF intensity (18, 24, and 30 kA/m). *N =* 3 per group. (C) Estimation of CEM_43_ values in response to MNP/AMF hyperthermia as a function of MNP concentration (1–3 mg/mL) and AMF intensity (18, 24, and 30 kA/m). The dotted red line indicates the reported threshold of the safe thermal dose for the application of hyperthermia to skin tissue [20]. *N* = 3 per group.

The threshold for thermal damage to human tissue in response to various hyperthermia treatments has been assessed by determining CEM_43_, an accepted metric for thermal dose assessment in a variety of human tissues [20,21]. In view of this, we first sought to determine the range of MNP/AMF parameters (MNPs concentration and AMF field intensity) that can be potentially applied for skin tissue within the safety margin for thermal dose. This was performed by correlating MNP/AMF-induced thermal doses with the values of CEM_43_ in the *in vitro* cell culture setting. For this, varying combinations of MNPs concentration (1–3 mg/mL) and AMF strength (18–30 kA/m) were applied to the culture of *S. aureus* biofilm and the change in ambient temperature (ΔT) in the media of *S. aureus* biofilm was measured (Figure 1(A)). It was previously shown that MNPs dose of up to 3 mg/mL had no direct cytotoxic effect on mammalian cells [22] and thus the maximum dose of MNPs used in this study was limited to 3 mg/mL as a threshold concentration nontoxic to the host cells. The change in temperature within the wells of *S. aureus* biofilm during MNP/AMF application was strongly dependent on the concentration of MNPs as well as intensity of AMF applied (Figure 1(B)). The application of AMF at a field strength of 18 kA/m resulted in ΔT of 5 °C at 1 mg/mL MNP for the given exposure time of 6 min and the ΔT further increased with increasing AMF intensity and MNPs concentration, achieving a ΔT of 19 °C at the 3 mg/mL-MNPs and 30 kA/m-AMF.

We next sought to correlate our measured values of ΔT induced by MNP/AMF hyperthermia with CEM_43_ for varying conditions of MNPs dose and AMF intensity. The estimated values of CEM_43_ were ranged from 1 min for 1 mg/mL of MNPs at 18 kA/m of AMF to as high as 7,500 min for 3 mg/mL of MNP at 30 kA/m of AMF (Figure 1(C)). Considering the CEM_43_ of 41 min as a threshold of the safe thermal dose for the application of hyperthermia to skin tissue [20], treatment conditions of MNP/AMF at up to 2 mg/mL-MNPs and 24 kA/m-AMF (equivalent to CEM_43_ of 34 min) or up to 1 mg/mL MNPs and 30 kA/m-AMF (equivalent to CEM_43_ of 5.7 min) were determined to be an acceptable range of thermal dose.

### MNP/AMF hyperthermia alone reduced the viability of S. aureus biofilm via manners dependent on MNP concentration and AMF intensity

Different bacterial strains have a varying degree of susceptibility to heat shock [23]. Thus, we first tested the thermo-susceptibility of *S. aureus* strain used in this study using a thermobiogram assay [24]. Our result shows that the bacterial strain is thermo-susceptible for both planktonic and biofilm phases, but somewhat less sensitive in biofilm phase than planktonic one (Supplementary Figure S1). Since metal oxide nanoparticles including iron oxide nanoparticles are capable of exhibiting antimicrobial effects by triggering an oxidative stress to the bacterial cells [25], we next assessed the effect of MNPs alone on the viability of *S. aureus* biofilm. The incubation of MNPs to the culture of *S. aureus* biofilm for 2 h at 3 mg/mL concentration reduced the CFU of *S. aureus* biofilm slightly by 35% (*p* < .05) in the absence of AMF, while MNPs at either 1 or 2 mg/mL concentration had no effect on their viability (*p* > .05) (Supplementary Figure S2).

**Figure 2.**
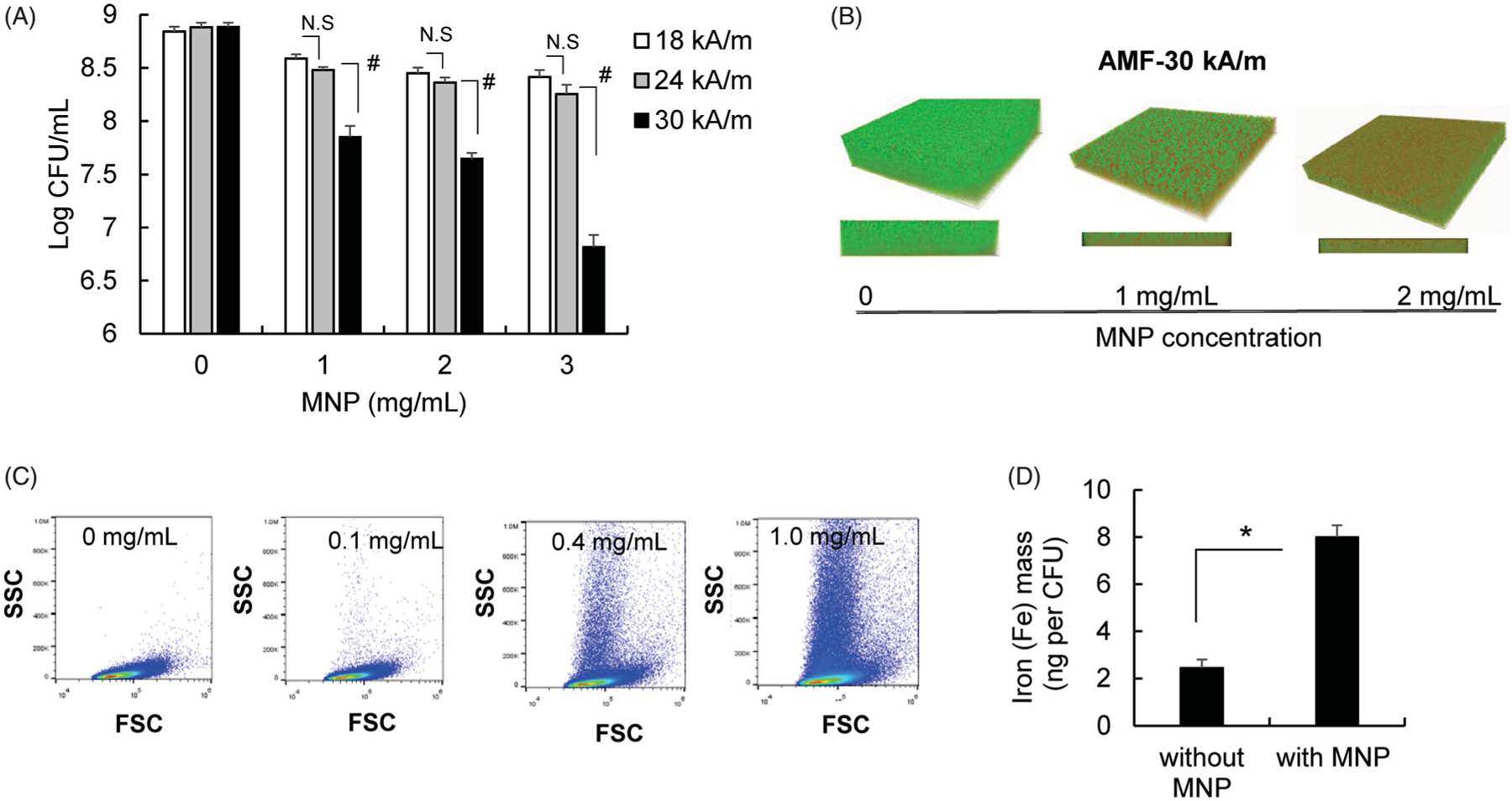
Efficacy of MNP/AMF hyperthermia alone on the killing of *S. aureus* biofilm bacteria. (A) The viable CFU numbers of *S. aureus* in the biofilm following MNP/AMF application as a function of MNPs concentration (1–3 mg/mL) and AMF intensity (18, 22, and 30 kA/m). *N =* 5–8 per group. N.S: Not Significant (*p* > .05). #: *p* < .01. (B) Representative SYTO9/PI fluorescence images following the application of MNP/AMF for varying concentrations of MNPs at 30 kA/m of AMF (green/SYTO9 live cells; red/PI = dead cells). Representative of three independent experiments. (C) Representative flow cytometric plots of forward and side scattering from *S. aureus* biofilm incubated with varying concentrations of MNPs (0–1 mg/mL) for 2 h. Representative of three independent experiments. (D) The atomic absorption spectroscopy analysis to quantify levels of intracellular iron in the *S. aureus* bacteria incubated with MNPs (1 mg/mL). *N* = 3 per group. *p < .05.

We next examined whether treatment of MNP/AMF hyperthermia with a safe thermal dose for MNP dosing and AMF strength was sufficient enough to eliminate bacterial biofilm. The application of MNP/AMF hyperthermia at the high thermal dose of 3 mg/mL-MNP and 30 kA/m-AMF (equivalent to CEM_43_ of 7,500 min) eradicated ~99% (2-log reduction) of *S. aureus* in the biofilm (Figure 2(A)). However, its efficacy become modest (<1-log reduction) when MNP/AMF was applied at below the CEM_43_ threshold thermal dose for skin tissue. For example, the application of MNP/AMF to *S. aureus* biofilm for 6 min at 1 mg/mL-MNP and 30 kA/m-AMF or at 2 mg/mL-MNP and 24 kA/m-AMF, which were deemed to be within the safe threshold of CEM_43_ for skin tissue, resulted in the reduction of bacterial CFUs by 88% and 65%, respectively. This was further confirmed by fluorescence images of live and dead staining of biofilms (Figure 2(B)). Our subsequent assay by flow cytometry revealed the presence of MNPs internalized by bacterial cells, as evidenced by increased side scattering patterns with increasing doses of MNPs (Figure 2(C)). The internalization of MNPs by *S. aureus* was further confirmed by the increased levels of iron in the bacterial cells, as quantified by AAS (Figure 2(D)). The application of direct heat shock to the *S. aureus* biofilm at a ΔT of 13 °C for 6 min exposure had no effect on the viability of *S. aureus* biofilm (Supplementary Figure S3), whereas a MNP/AMF-induced heat shock at 2 mg/mL-MNP and 30 kA/m-AMF, resulting in an increase of ΔT similar to the direct heat shock, significantly decreased the CFU number of biofilm bacteria (>1-log reduction, Figure 2(A)). Taken together, our results suggest that the heat shock generated by MNPs in the bacterial cell contributed to a decrease in bacterial cell viability following the exposure of MNP/AMF.

### Mild MNP/AMF hyperthermia synergistically enhanced the antibiotic susceptibility of S. aureus biofilm by facilitating the uptake of antibiotics

**Figure 3.**
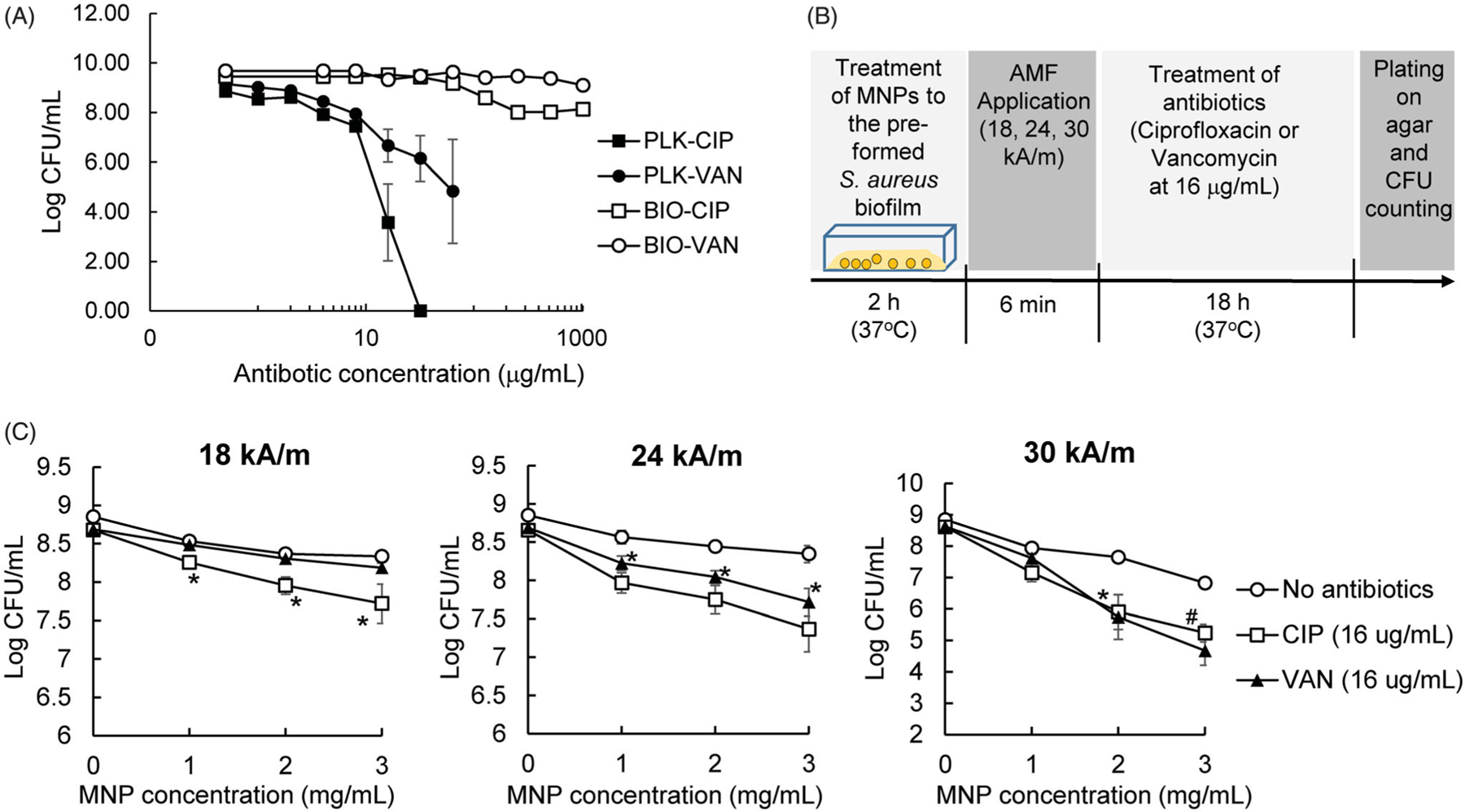
Efficacy of MNP/AMF hyperthermia on the susceptibility of *S. aureus* biofilm to antibiotics. (A) The susceptibility of planktonic (PLK) and biofilm (BIO) phase of *S. aureus* to ciprofloxacin (CIP) and vancomycin (VAN). *N =* 4 per group. (B) A schematic diagram for the experimental protocol to quantify the effect of MNP/AMF on the susceptibility of *S. aureus* biofilm to antibiotics. (C) The effect of MNP/AMF hyperthermia on the susceptibility of *S. aureus* biofilm to CIP and VAN for varying conditions of MNP concentration (1, 2, 3 mg/mL) and AMF intensity (18, 24, 30 kA/m). *N =* 5–9 per group. *p < .05 and #*p* < .01 vs No antibiotics group.

By observing the modest efficacy of MNP/AMF hyperthermia in killing biofilm bacteria when applied below the threshold of a safe thermal dose, we next examined whether a combined application of MNP/AMF hyperthermia with antibiotics would enhance anti-biofilm efficacy. The susceptibility of planktonic and biofilm phase *S. aureus*, formed by the same number of bacterial cells (2 × 10^8^ CFU/mL), to antibiotics CIP or VAN was evaluated first. The treatment of CIP or VAN at a dose of 16 µg/mL exhibited bactericidal effects against planktonic *S. aureus*, as observed by reduction in the viability of the bacteria by 5-log and 3-log, respectively (Figure 3(A)). However, the treatment of CIP or VAN at the same dose of 16 µg/mL had no effect on the viability of *S. aureus* in the biofilm phase. The viability of *S. aureus* biofilm decreased by 1-log at 128 µg/mL without further reduction up to 1,024 µg/mL for CIP, and by only 1-log at 1,024 µg/mL for VAN (Figure 3(A)). The application of MNP/AMF at 2 mg/mL-MNP and 30 kA/m-AMF for 6 min, followed by the exposure of either CIP or VAN at 16 µg/mL resulted in a 2-log reduction of *S. aureus* biofilm, while the application of MNP/AMF alone resulted in less than 1-log reduction in biofilm cell viability (Figure 3(B,C)). Since the therapeutic efficacy of combined treatment is greater than the sum of the efficacy of their individual treatments, our results suggest that the application of mild MNP/AMF hyperthermia could provide a synergistic effect when combined with a clinically relevant dose of conventional antibiotics.

**Figure 4.**
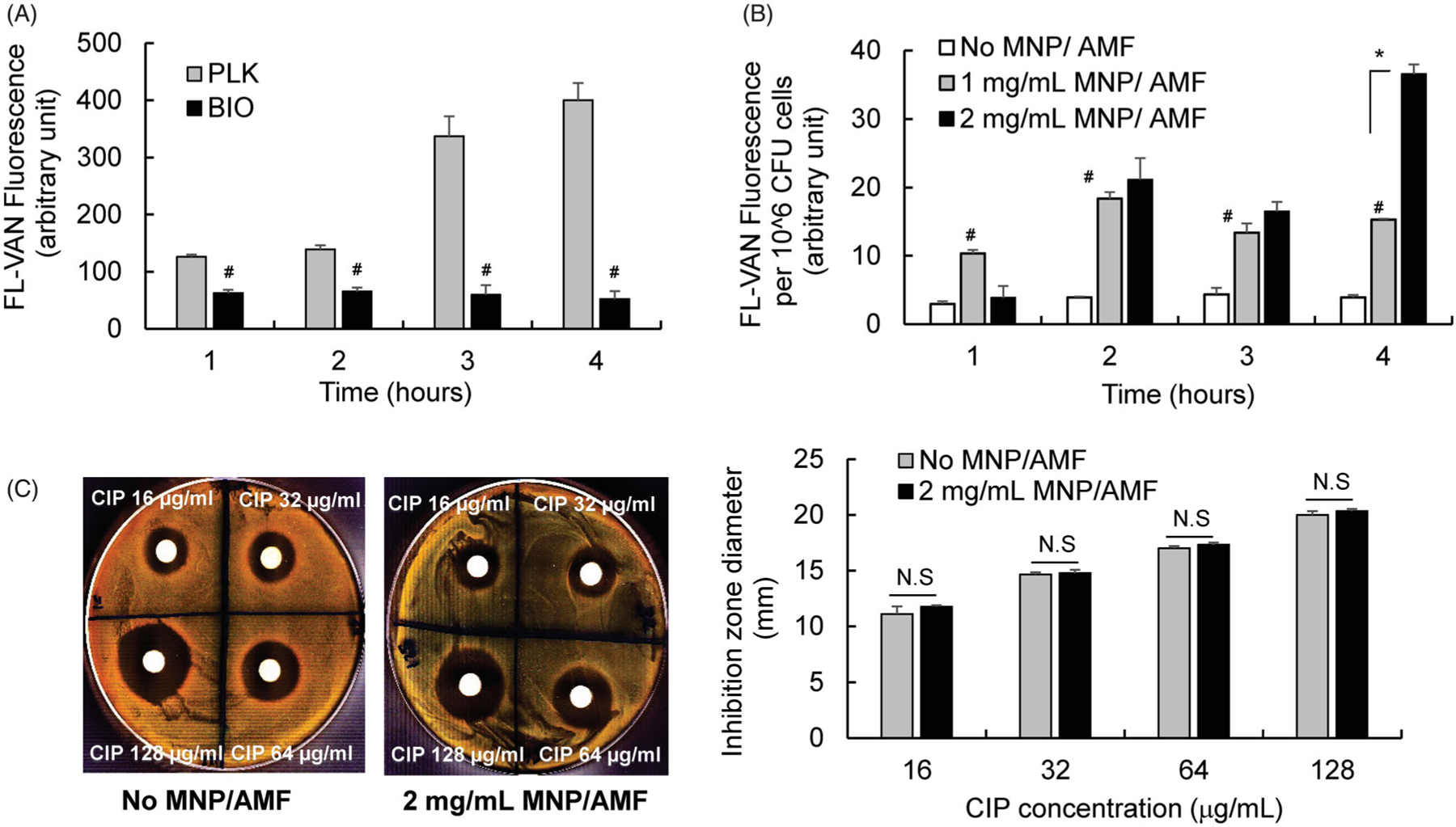
Effects of MNP/AMF hyperthermia on the uptake of antibiotics by *S. aureus* biofilm. (A) The uptake of FL-VAN by planktonic and biofilm phase of S. *aureus*, quantified by fluorescence emitted from FL-VAN in the bacterial cells. *N =* 6 per group. #*p* < .01 vs PLK group. (B) The effect of MNP/AMF hyperthermia on the uptake of FL-VAN by *S. aureus* biofilm. The fluorescence emitted from FL-VAN in the *S. aureus* collected from biofilm culture for varying concentrations of MNPs (0–2 mg/mL) in the absence and presence of AMF application (30 kA/m). *N =* 6 per group. #*p* < .01 vs No MNP/AMF group. *p < .01. (C) The penetration of CIP (16, 32, 64, and 128 µg/mL) through the *S. aureus* biofilm in the absence or presence of MNP/AMF application (pre-exposure at 2 mg/mL-MNPs and 30 kA/m-AMF for 6 min) assessed by disk diffusion method. Left: Representative photographic images of TSA plate showing the zone of growth inhibition by CIP. Right: Quantification for the diameter of the zone of growth inhibition by CIP. *N* = 3 per group. N.S: Not Significant (*p* > .05).

Our subsequent experiments for testing antibiotic uptake using FL-VAN revealed that the synergistic effect between mild MNP/AMF hyperthermia and antibiotics was associated with an increased uptake of antibiotics by bacterial cells in the biofilm. The uptake of FL-VAN by *S. aureus* in the biofilm phase was significantly attenuated compared to the planktonic *S. aureus* (Figure 4(A)). The application of mild MNP/AMF (at 1 mg/mL of MNP and 30 kA/m of AMF) significantly increased the uptake of FL-VAN (Figure 4(B)). It has been suggested that the presence of EPS or extracellular DNA in the biofilm may result in reduced exposure of biofilm bacteria to antibiotics by physically restricting their penetration through the biofilms or acting as an absorbent or reactant of antibiotics [26,27]. Additionally, it was previously shown that nanoparticle-induced hyperthermia could enhance the transport of the antibiotics by dispersing the biofilm matrix [28]. However, our results from antibiotic penetration assay using a disk diffusion method demonstrated that the diffusion of CIP through the *S. aureus* biofilm was not significantly altered by the application of MNP/AMF hyperthermia (*p* > .05), as quantified by the diameter of the zone of growth inhibition (Figure 4(C)). Taken together, our results suggest that the diminished susceptibility of *S. aureus* biofilm to antibiotics might be due to an attenuated uptake of antibiotics by biofilm bacteria, which could be reversed by MNP/AMF hyperthermia-induced heat shock.

### Mild MNP/AMF hyperthermia augmented the bactericidal activity of macrophages against intracellular S. aureus

**Figure 5.**
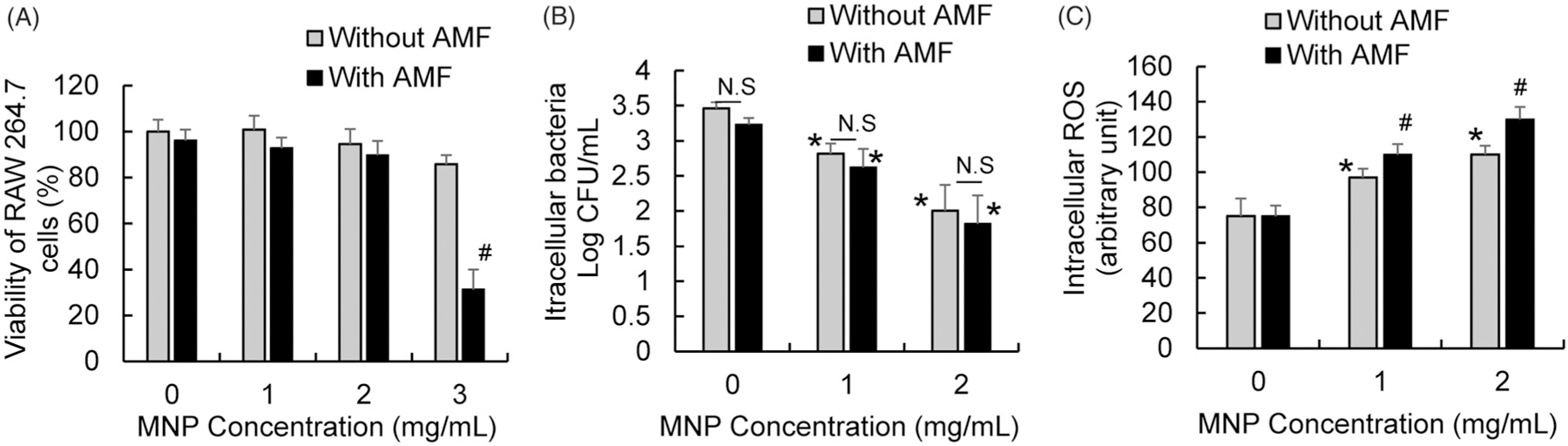
Effects of MNP/AMF hyperthermia on the viability, bactericidal activity, and ROS generation of RAW 264.7 macrophages. (A) The viability of RAW 264.7 macrophages for varying concentrations of MNPs (0–3 mg/mL) in the absence or presence of AMF application at the intensity of 30 kA/m, assessed by MTT viability assay. *N =* 4 per group. (B) The effect of varying concentrations of MNPs (0–2 mg/mL) on the bactericidal activity of RAW 264.7 cells in the absence and presence of AMF application (30 kA/m), assessed by antibiotic protection assay. *N =* 10–15 per group. (C) The effect of varying concentrations of MNPs (0–2 mg/mL) on the ROS generation in RAW 264.7 cells in the absence and presence of AMF application (30 kA/m). *N =* 4–6 per group. N.S: Not Significant (*p* > .05). *p < .05 vs untreated control (without MNP/AMF). #*p* < .05 vs without AMF group at a given MNP concentration.

In response to tissue injury and infection, the innate immune response, associated with the phagocytic and bactericidal activities of macrophages, plays a critical role in host defense [29]. To effectively apply MNP/AMF hyperthermia as an anti-biofilm therapy, it should therefore be evaluated whether this therapy aids or detracts from host innate immune response. In view of this, we evaluated the impact of MNP/AMF hyperthermia on the bactericidal activity of macrophages, a critical host defense mechanism against infectious challenges [30,31]. We first assessed the cytotoxicity of MNP/AMF hyperthermia on RAW 264.7 macrophages at the same thermal doses used in the biofilm experiments above. The application of MNP/AMF hyperthermia at a high thermal dose (at 3 mg/mL-MNP and 30 kA/m-AMF) significantly decreased the viability of RAW 264.7 macrophages by ~70% (Figure 5(A)). However, cell viability for other conditions of MNPs concentration and AMF intensity was not significantly altered, compared to untreated control. The capacity of RAW 264.7 macrophages to kill intracellular bacteria was assessed by using an antibiotic protection assay, which enables the quantification of the number of surviving bacteria within macrophages [32]. The number of viable intracellular *S. aureus* was significantly decreased by 85% at 1 mg/mL MNPs compared to untreated control, and it was further decreased with increasing concentration of MNPs to 2 mg/mL at the fixed AMF intensity of 30 kA/m (Figure 5(B)). However, there was no significant difference in bactericidal activity between macrophages with and without AMF application at the same MNP dose (Figure 5(B)), suggesting that exposure of MNPs to the RAW 264.7 macrophages was sufficient enough to exhibit a bactericidal activity against intracellular *S. aureus*. Since iron oxide magnetic nanoparticles can trigger the generation of ROS [33–35], and ROS can exhibit an antimicrobial activity [36], we next examined whether the improved bactericidal activity of macrophages with MNPs alone or MNP/AMF hyperthermia was associated with increased ROS generation. The generation of intracellular ROS in the RAW 264.7 cells was significantly increased with increasing concentration of MNPs (Figure 5(C)). Interestingly, the application of AMF further increased ROS generation in RAW 264.7 macrophages, suggesting that, in addition to MNP-triggered ROS generation, heat stress induced by MNP/AMF hyperthermia might contribute to ROS generation.

## Discussion

**Figure 6.**
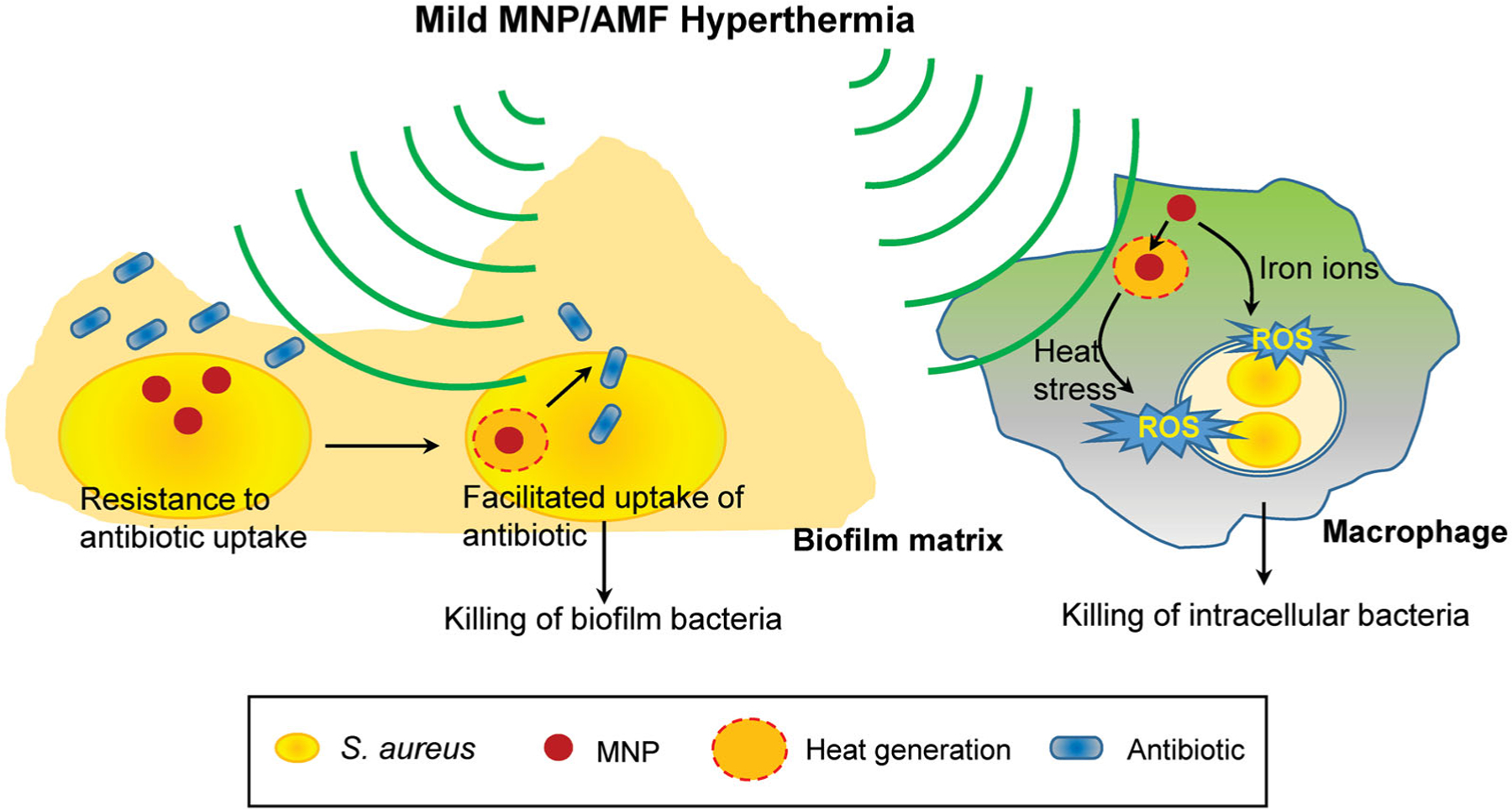
A schematic depicting the therapeutic potential of mild MNP/AMF hyperthermia against biofilm infections. The application of mild MNP/AMF hyperthermia can enhance the susceptibility of *S. aureus* biofilm to antibiotics by facilitating their uptake to biofilm bacteria. Additionally, when applied to macrophages at lower thermal doses, the treatment of MNP/AMF can promote a macrophage-mediated host response for the clearance of intracellular bacteria *via* MNP-dependent generation of ROS.

Recently, several reports including ours have demonstrated that MNP/AMF hyperthermia therapy can be an effective approach to reduce the viability of bacterial pathogens [9,28,37,38]. However, despite its therapeutic potential, the use of MNP/AMF hyperthermia alone for the purpose of treating infection has been limited due to a potential risk for off-target thermal damage to the host tissue [39], associated with a high thermal dose necessary to attain sufficient bacterial clearance. The major finding of this study is that the effective application of MNP/AMF hyperthermia within a safe thermal dose threshold is feasible to use against *S. aureus* biofilm when combined with conventional antibiotics. Additionally, MNP/AMF hyperthermia at the safe thermal dose can aid host innate immune response for the clearance of intracellular bacteria (Figure 6).

The effect of heat shock on the susceptibility of bacterial biofilms to antibiotics has been studied in experimental systems that induce uniformly increased temperature to culture media [6,13]. For example, the application of heat shock at a ΔT of 13 °C for 30 min or a ΔT of 23 °C for 5 min exhibited synergistic effects in eradicating *P. aeruginosa* biofilm when combined with antibiotics including ciprofloxacin or tobramycin [28]. A similar phenomenon of synergism between heat shock and antibiotics was observed in bacterial biofilms formed by gram positive *Staphylococcus epidermis*, and to a somewhat lesser extent in *S. aureus*, under heat shock conditions at a ΔT of 8 °C for 120 min [14]. However, in each of these conditions, the estimated CEM_43_ values would be much higher than the reported threshold for thermal damage for skin tissue [18,21]. It has been shown that heat treatment could induce acute damage to skin function at a CEM_43_ of 41 min [21], immediate superficial burns at CEM_43_ of between 480 and 960 min [40], and necrosis of human skin at CEM_43_ of between 288 and 1.5 × 10^4^ min [21].

Our results demonstrate that mild MNP/AMF hyperthermia is an effective strategy that can render drug-resistant biofilm bacteria to become susceptible to conventional antibiotics. This appears to be associated with the capacity of MNP/AMF to generate localized heat shock inside and/or in the vicinity of the bacterial cells. The treatment of antibiotics, CIP or VAN, at a bactericidal dose for planktonic *S. aureus* (16 µg/mL, Figure 3(A)), had little to no effect on the viability of *S. aureus* biofilm. However, a bactericidal effect was observed when the biofilms were pre-exposed to MNP/AMF hyperthermia at a thermal dose below the CEM_43_ threshold for skin tissue, suggesting a synergistic efficacy between MNP/AMF hyperthermia and antibiotics. Since the treatment of bacterial biofilms requires a high dose of antibiotics with sustained administration [41], which can lead to overdose side effects, the combined treatment of MNP/AMF hyperthermia with antibiotics can be an effective strategy at reduced antibiotic doses.

Although a detailed mechanism for the synergism between MNP/AMF hyperthermia and antibiotics remains to be elucidated, our results suggest that this is associated with the enhanced uptake of antibiotics by bacterial cells, with little effect by the penetration of antibiotics through the biofilm matrix. This is in line with several recent studies suggesting that decreased antibiotic penetration through the biofilm matrix may not be the main cause of antibiotic resistance of bacterial biofilms [42,43]. One possible explanation for the observed synergism is that an increase of localized temperature by MNP/AMF might result in an increased metabolic activity in bacterial cells, which in turn, facilitate the uptake of antibiotics by biofilm bacteria. This may be supported by the findings of transcriptomic and metabolic data of *S. aureus* in response to an exposure of a sub-lethal (43 °C) temperature, which demonstrated that heat stress exposure could significantly increase metabolic activities including ATP-generating pathways [44]. Additionally, the stimulation of central metabolic pathway in drug-resistant bacteria was shown to significantly increase the uptake of antibiotics [45]. However, the detailed mechanism by which MNP/AMF hyperthermia facilitates antibiotic uptake remains to be elucidated.

Apart from its efficacy in sensitizing biofilm bacteria to an antimicrobial treatment, MNP/AMF hyperthermia may be beneficial in a macrophage-mediated innate immune response for bacterial clearance. Although the application of MNP/AMF at high thermal dose was detrimental to macrophage viability, suitable parameters for MNP concentration and AMF strength were found to cooperate with macrophage-mediated bacterial clearance. The improved bactericidal activity of macrophages in response to MNP/AMF hyperthermia was associated with increased generation of ROS by MNPs internalized by macrophages. Iron ions can impact the intracellular redox signaling for the generation of ROS inside cells *via* a Fenton reaction [46], essential components of the innate immune response against intracellular bacteria [36]. The presence of MNPs within macrophages may have similarly triggered an increase in ROS production by increasing the levels of intracellular iron (Supplementary Figure S4). It was shown that cellular ROS generation could be enhanced when cells with internalized MNPs were exposed to an AMF [47]. Consistent with this finding, the application of AMF in the presence of MNP could further increase ROS generation in RAW 264.7 macrophages compared to the cells without an AMF treatment. This, however, was not sufficient enough to result in improved bactericidal activity, suggesting that the beneficial effect of MNP/AMF hyperthermia on the host innate immune response is largely due to the ROS generated by internalized MNPs.

In summary, in response to a clear and present need for an effective antimicrobial therapy for biofilm infections, our findings demonstrate the feasibility of mild MNP/AMF hyperthermia as a minimally invasive strategy to treat biofilm infection, which has the potential to work synergistically with standard-of-care antibiotic treatment and the innate immune response to accelerate the resolution of biofilm infections.

## Supplementary Material

Supplementary Material

## Data Availability

The data that support the findings of this study are available from the corresponding author (MK) upon reasonable request.
