# Supplementary material for: Mild magnetic nanoparticle hyperthermia enhances the susceptibility of Staphylococcus aureus biofilm to antibiotics
Source: Int J Hyperthermia. Author manuscript; Available in PMC 2020 Dec 11. (PMC7730973; doi:10.1080/02656736.2019.1707886)
Supplement: Supplementary Material [file NIHMS1641496-supplement-Supplementary_Material.docx]

**Supplemental Figures**


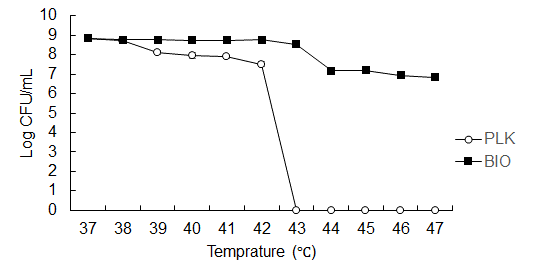


**Fig. S1**. Thermobiogram analysis for thermal sensitivity of planktonic (PLK) and biofilm (BIO) phase of *S. aureus*. The planktonic and biofilm phase of *S. aureus* were incubated at varying temperature levels (e.g., 37 °C, 38 °C, 39 °C, 40 °C, 41 °C, 42 °C, 43 °C, 44 °C, 45 °C, 46 °C, 47 °C) for 24 h under shaking and static culture conditions, respectively. Then, the cells were plated on TSA for bacterial CFU counting. N= 6 per group.


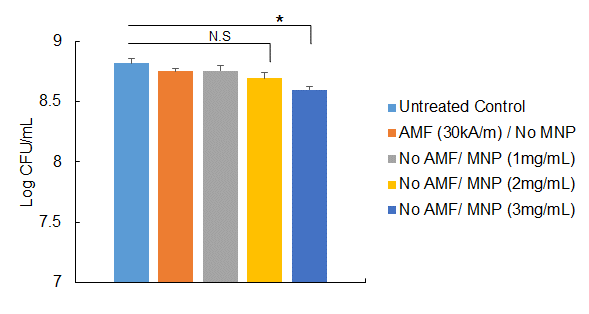


**Fig. S2**. The effect of MNPs (1, 2, or 3 mg/mL) or AMF (30 kA/m) alone on the viability of *S. aureus* biofilm. **AMF (30kA/m)/No MNP group**: The *S. aureus* biofilm pre-formed on an 8 well-chamber slide was applied with an AMF for 6 min at 30 kA/m. **No AMF/MNP groups**: The *S. aureus* biofilm pre-formed on an 8 well-chamber slide was incubated with MNPs at varying concentrations (1, 2, 3 mg/mL) for 2 h at 37 °C. The cells for all the groups were plated on TSA for bacterial CFU counting after disrupting the biofilm matrix with sonication. N=8 per group. *: *p*<0.05 vs untreated control group. N.S: Not significant (p>0.05).


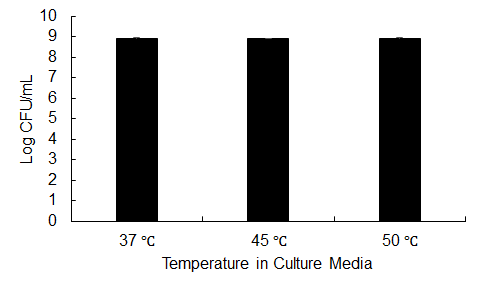


**Fig. S3**. The effect of direct heat shock on the on the viability of *S. aureus* biofilm. *S. aureus* biofilm were cultured on a tissue culture treated 48-well polystyrene plate and divided into three groups. Then, the cells were incubated at 37°C or elevated temperature of 45°C and 50°C for 6 min. The cells were then plated on TSA for bacterial CFU counting after disrupting the biofilm matrix with sonication. N=4 per group.


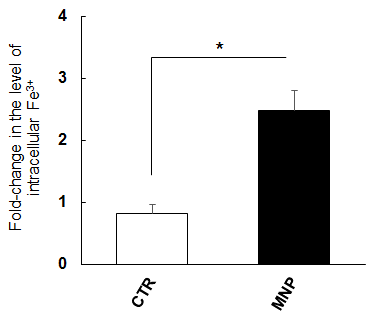


**Fig. S4**. The levels of intracellular iron ions (Fe^3+^) in RAW 264.7 macrophages following the exposure of MNPs. The intracellular level of iron in RAW 264.7 cells was measured using an iron assay kit (Abcam, Cambridge, MA). For this, RAW 264.7 cells were treated with either MNPs (3 mg/mL) or vehicle solution for 2 h and then homogenized in iron assay lysis buffer to measure the levels of Fe^3+^. The relative levels of Fe^3+^ were normalized to the untreated control group and expressed as a fold change. N=3 per group. *: p<0.05
